# Emerging Trends in the Epidemiology of COVID-19: The Croatian ‘One Health’ Perspective

**DOI:** 10.3390/v13122354

**Published:** 2021-11-24

**Authors:** Tatjana Vilibic-Cavlek, Vladimir Stevanovic, Diana Brlek-Gorski, Ivana Ferencak, Thomas Ferenc, Magdalena Ujevic-Bosnjak, Irena Tabain, Natasa Janev-Holcer, Ivana Perkovic, Mario Anticevic, Barbara Bekavac, Bernard Kaic, Anna Mrzljak, Marin Ganjto, Ljiljana Zmak, Maja Mauric Maljkovic, Pavle Jelicic, Lovro Bucic, Ljubo Barbic

**Affiliations:** 1 Department of Virology, Croatian Institute of Public Health, 10000 Zagreb, Croatia; ivana.ferencak@hzjz.hr (I.F.); irena.tabain@hzjz.hr (I.T.); 2 School of Medicine, University of Zagreb, 10000 Zagreb, Croatia; anna.mrzljak@gmail.com; 3 Department of Microbiology and Infectious Diseases with Clinic, Faculty of Veterinary Medicine University of Zagreb, 10000 Zagreb, Croatia; ljubo.barbic@vef.hr; 4 Environmental Health Department, Croatian Institute of Public Health, 10000 Zagreb, Croatia; diana.brlek-gorski@hzjz.hr (D.B.-G.); magdalena.ujevic@hzjz.hr (M.U.-B.); natasa.janev@hzjz.hr (N.J.-H.); ivana.perkovic@hzjz.hr (I.P.); mario.anticevic@hzjz.hr (M.A.); barbara.bekavac@hzjz.hr (B.B.); pavle.jelicic@hzjz.hr (P.J.); lovro.bucic@hzjz.hr (L.B.); 5 Clinical Department of Diagnostic and Interventional Radiology, Merkur University Hospital, 10000 Zagreb, Croatia; thomas.ferenc95@gmail.com; 6 Department of Social Medicine and Epidemiology, Faculty of Medicine, University of Rijeka, 51000 Rijeka, Croatia; 7 Department of Epidemiology, Croatian Institute of Public Health, 10000 Zagreb, Croatia; bernard.kaic@hzjz.hr; 8 Department of Gastroenterology and Hepatology, University Hospital Center Zagreb, 10000 Zagreb, Croatia; 9 Zagreb Wastewater-Management and Operation Ltd., 10000 Zagreb, Croatia; marin.ganjto@zovuip.hr; 10 Department for Tuberculosis, Croatian Institute of Public Health, 10000 Zagreb, Croatia; ljiljana.zmak@hzjz.hr; 11 Department for Animal Breeding and Livestock Production, Faculty of Veterinary Medicine, University of Zagreb, 10000 Zagreb, Croatia; maja.mauric@vef.hr

**Keywords:** COVID-19, SARS-CoV-2, variants, humans, pet animals, wildlife, environment, Croatia

## Abstract

During the four pandemic waves, a total of 560,504 cases and 10,178 deaths due to COVID-19 were reported in Croatia. The Alpha variant, dominant from March 2021 (>50% of positive samples), was rapidly replaced by Delta variants (>90%) by August 2021. Several seroprevalence studies were conducted in different populations (general population, children/adolescents, professional athletes, healthcare workers, veterinarians) and in immunocompromised patients (hemodialysis patients, liver/kidney transplant recipients). After the first pandemic wave, seroprevalence rates of neutralizing (NT) antibodies were reported to be 0.2–5.5%. Significantly higher seropositivity was detected during/after the second wave, 2.6–18.7%. Two studies conducted in pet animals (February-June 2020/July–December 2020) reported SARS-CoV-2 NT antibodies in 0.76% of cats and 0.31–14.69% of dogs, respectively. SARS-CoV-2 NT antibodies were not detected in wildlife. Environmental samples taken in the households of COVID-19 patients showed high-touch personal objects as most frequently contaminated (17.3%), followed by surfaces in patients’ rooms (14.6%), kitchens (13.3%) and bathrooms (8.3%). SARS-CoV-2 RNA was also detected in 96.8% affluent water samples, while all effluent water samples tested negative. Detection of SARS-CoV-2 in humans, animals and the environment suggests that the ‘One Health’ approach is critical to controlling COVID-19 and future pandemics.

## 1. Introduction

The ongoing global epidemic caused by severe respiratory syndrome coronavirus 2 (SARS-CoV-2) demonstrated the vulnerability of both humans and animals to the threat posed by coronaviruses. At the end of 2019, numerous cases of uncommon viral pneumonia started to emerge in Wuhan City, China [1]. Due to its efficient transmissibility, the spread of the novel virus resulted in outbreaks of identical cases across China. Tentatively, it was referred to as new coronavirus 2019 (2019-nCoV) and, at the beginning of February 2020, was renamed SARS-CoV-2; the consequent disease was named coronavirus disease 2019 (COVID-19) [2]. The continuous worldwide spread initiated World Health Organization to declare COVID-19 a pandemic in March 2020 [3]. Modes of transmission of this newly emerged virus are still being studied. Like some other coronaviruses, SARS-CoV-2 is mainly transmitted through respiratory and airborne pathways; however, other modes of transmission were also reported [4]. It is assumed that the origin of SARS-CoV-2 is similar to SARS-CoV and Middle East respiratory syndrome coronavirus (MERS-CoV), which had bat species as natural hosts and gained access to the human population through the intermediate animal host [5,6,7]. The zoonotic potential adds another dimension in understanding the epidemiology of coronaviruses and successful surveillance. It is now known that SARS-CoV-2 can infect several wild and domestic animals [8,9,10]. However, the role of animals in the epidemiology of COVID-19 and the influence of SARS-CoV-2 on animal health have not been fully understood.

This article aims to sum up the data on various aspects of SARS-CoV-2 epidemiology in Croatia. It brings together studies on the SARS-CoV-2 prevalence and molecular epidemiology in the human population, wild/domestic animals and the environment (‘One Health’ concept).

## 2. COVID-19 in Humans

**Figure 1:**
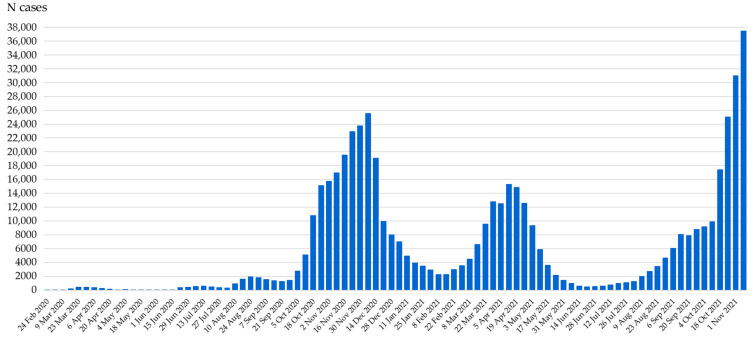
COVID-19 pandemic waves in Croatia (weekly detected SARS-CoV-2 cases, February 2020–November 2021).

The first case of COVID-19 in Croatia was reported on 25 February 2020 in a traveler returning from Milan, Italy. So far, 560,504 of cases and 10,178 deaths due to COVID-19 have been reported during the four pandemic waves (Figure 1) [11].

Several seroepidemiological studies were conducted in different exposed and non-exposed population groups during/after the first and second pandemic waves (Table 1).

A study conducted in the Croatian general population showed a significant difference in the seroprevalence of SARS-CoV-2 after the first (May–July 2020) and second (December 2020–February 2021) pandemic waves. The overall prevalence of binding antibodies (ELISA) and neutralizing (NT) antibodies (VNT) after the first wave was lower (ELISA 2.2%, VNT 0.2%) compared to the second wave (ELISA 25.1%, VNT 18.7%). Seropositive individuals were detected in all age groups. SARS-CoV-2 NT antibody titers during/after the second wave seemed to be age-related with the highest NT activity in children under 10 years and individuals above 50 years. Some studies have shown that SARS-CoV-2 NT antibody responses are more robust in patients with severe disease [19]; therefore, higher NT antibody titers in the elderly could be explained by more severe symptoms in this population group. A possible explanation for higher antibody levels in children could be cross-immunization by previous exposures to seasonal coronaviruses, since epitopes for T and B cells were found to be conserved among SARS-CoV-2, HCoV-OC43 and HKU1, which also contribute to children’s relative protection from SARS-CoV-2 infection [20].

Children and adolescents were tested at three time points: May 2020 (first wave), October–November 2020 (peak of the second wave) and December 2020–February 2021 (end of the second wave). The VNT seroprevalence rate in the pediatric population differed with 2.9%, 8.4% and 19.0% seropositive participants [14,17]. While there was no difference in the seropositivity after the first wave, this population group showed a lower prevalence rate compared to the general adult population after the second wave [17]. It is worth noting that public health measures were less restrictive at the beginning of the second wave in Croatia, with children returning back to schools; the lower odds for seropositivity can be attributed to the lower susceptibility of children to SARS-CoV-2 infection, as previously suggested [21]. Comparison of the seroprevalence in children/adolescents and the general population during the first wave further supported the age-dependent difference in the VNT seropositivity. Although there was no difference in the ELISA positivity (3.9% vs. 2.2%), it was confirmed that younger individuals developed measurable NT antibodies more often (2.9% vs. 0.2%; Table 1). 

While there was a significant difference in the prevalence of binding antibodies between inhabitants of continental and coastal counties (26.8% and 21.9%, respectively), the prevalence of NT antibodies did not differ significantly among regions (19.8% and 16.6%, respectively) [17]. 

Sporting events can present a risk during the COVID-19 pandemic, since social distancing in this context is not always possible [22]. During the first pandemic wave (June 2020), professional athletes had significantly higher seroprevalence than the general population tested at the same period (ELISA 11.1% vs. 2.2%; VNT 5.5% vs. 0.2%). Even though social distancing and travel bans were in place in Croatia, international travel is an essential part of a competition for many athletes, placing these groups at risk of COVID-19. More than half of participants (51.1%) reported traveling abroad (Europe 36.7%; Europe/Australia 5.6%; the USA 4.4%; Asia 2.2%; and Africa 2.2%) and 18.9% reported COVID-19 related symptoms in the past six months. Risk analysis showed that professional athletes were 5.54 times more likely to test positive by ELISA and 31.94 times more likely to test positive by VNT than the general population (Table 2).

Several studies showed that a large number of outbreaks and a high incidence rate of outbreak-associated COVID-19 cases occurred in manufacturing [23,24]. In April 2020, factory employees (DIV company specialized in the production and trade of screws and other mechanical parts, metal products and shipbuilding) from two Croatian coastal counties were screened for SARS-CoV-2 IgG antibodies. Using the immunochromatography test, 1.27% of participants were seropositive [12]. Compared to the general population, there was no evidence of increased viral exposure in the manufacturing sector (Table 2), probably due to the hard lockdown measures. 

Aside from manufacturing, construction was a common sector for workplace outbreaks in other countries [25]. In May 2020, a group of construction workers was tested for SARS-CoV-2 antibodies. On 22 March 2020, during the COVID-19 epidemic, an earthquake of magnitude 5.3 hit Zagreb, the capital of Croatia, causing many buildings to be extensively damaged or out of use. In total, 135 volunteer construction workers working on damaged buildings in Zagreb and its surroundings were studied. Although they were at risk of COVID-19 since they worked directly with the people after social distancing regulations to prevent the spread of coronavirus had been put in place, a low overall seroprevalence rate (2.9% ELISA-positive, 2.2% VNT-positive) indicated that the spread of the epidemic did not accelerate after the earthquake [15]. However, risk analysis suggested that construction workers were 12.34 times more likely to test VNT-positive, suggesting increased exposure to SARS-CoV-2 (Table 2). 

During April and May 2020, 592 serum samples from healthcare workers (HCWs) and allied/auxiliary HCWs were tested for the presence of SARS-CoV-2 antibodies. Convenient samples were collected from six continental and coastal counties with a high incidence of COVID-19. Using ELISA, SARS-CoV-2 IgG antibodies were detected in 2.7% of participants, while NT antibodies were detected in 1.5%. Seven seropositive individuals were healthcare professionals, while two were administrative workers. All but one HCWs worked in the infectious disease department [13]. ELISA seropositivity rate was not higher than in the general population (2.9% vs. 2.2%), but NT antibodies were recorded more frequently (1.5% vs. 0.2%), indicating a higher risk of infection due to frequent exposure to SARS-CoV-2.

Owing to the hypothesized zoonotic origin of SARS-CoV-2, in May 2020, 122 serum samples from employees of the Faculty of Veterinary Medicine University of Zagreb were collected. Using ELISA, 5.19% of administrative, basic and pre-clinical sciences department personnel and 5.13% of animal health service providers and laboratory personnel tested SARS-CoV-2 IgG positive. However, NT antibodies were not detected in tested samples [16]. There was no difference in the ELISA positivity rate between groups, suggesting that pet animals had no significant role in spreading infection. In March 2021, second testing was performed, which included 121 veterinary personnel. Seroprevalence was significantly higher compared to the first sampling. Seropositivity was 18.2% using ELISA, while 9.1% had SARS-CoV-2 NT antibodies [15]. The higher seroprevalence in veterinarians (ELISA) compared to the Croatian general population (4.9% vs. 2.2%) during the first wave may have been the result of veterinary practice, which implies numerous close contacts with animal owners (e.g., stepping in to help restrain an animal). However, there was no difference in the prevalence of NT antibodies. On the other hand, lower VNT seropositivity (9.1% vs. 18.7%) during the second wave may be due to a smaller sample size or more efficient SARS-CoV-2 spread in the general population during the pandemic.

Whether solid organ transplant recipients (SOTRs) are at increased risk for severe COVID-19 compared with the general population is controversial [26]. From September to November 2020 (beginning of the second wave), a cross-sectional screening for COVID-19 among 512 adult outpatient liver and kidney transplant recipients was performed. The transplanted cohort’s seroprevalence was 20.1% by ELISA and 3.1% by VNT. NT antibodies were detected in 15.6% of anti-SARS-CoV-2 ELISA IgG-positive SOTRs. Overall VNT positivity rates were higher in patients who reported participation in large community events (5.9% vs. 1.1%; *p* = 0.027), while no significant difference was detected in the seroprevalence rate regarding received blood products (3.0% vs. 1.6%; *p* = 0.553) and travelling habits (5.3% vs. 1.1%; *p* = 0.085). In addition, symptomatic VNT-positive patients showed significantly higher NT antibody titers (median 128, IQR = 32–128) compared to asymptomatic patients (median 16, IQR = 16–48) [18]. Compared to the general population tested after the first wave, the prevalence of NT antibodies was slightly higher in SOTRs (3.1% vs. 2.2%), demonstrating the development of protective immunity despite impaired immunological status. However, it is important to note that ELISA-positive liver transplant recipients were 4.39 (95% CI OR = 2.21–8.74, *p* < 0.001) times less likely and kidney transplant recipients were 5.46 (95% CI OR = 2.29–13.0, *p* < 0.001) less likely to test VNT-positive compared to the general population.

In conclusion, serologic surveys showed that the SARS-CoV-2 seroprevalence differed according to the sampling time and population groups. Temporal trends in the seroprevalence followed the COVID-19 pandemic waves in Croatia.

## 3. COVID-19 in Pet Animals

The first large-scale serosurvey of SARS-CoV-2 in dogs and cats from two regions (Zagreb and Split) in Croatia was conducted from February to June 2020. Zagreb (continental region) and Split (coastal region) were selected as the two cities with the highest number of human COVID-19 cases during the first pandemic wave in Croatia. The first dog and cat serum samples with positive VNT were collected in April in Zagreb, a few weeks after the first COVID-19 human case in the same area. Even though the number of samples with NT antibodies was low, it is worth mentioning that the difference in the prevalence in dogs (0.31%) and cats (0.76%) was not statistically significant. In the study, 172 dog samples were also tested using ELISA. The seroprevalence of SARS-CoV-2 among dogs at the end of the first wave was 7.56%, with the highest number of positive samples collected six weeks after the peak in the number of human cases [16]. 

The second study, conducted from July to December 2020, followed the seroprevalence of SARS-CoV-2 infection in two dog populations during most of the second wave in Zagreb. The first group included dogs that shared households with confirmed human COVID-19 cases (N = 78). The general population was represented by dogs admitted to the Veterinary Teaching Hospital (Faculty of Veterinary Medicine, University of Zagreb) for any given reason (N = 1069). In COVID-19-infected households, 43.9% of dogs tested ELISA-positive and 25.64% had detectable NT antibodies, values comparable with secondary attack rate in humans [27]. In the general population, the ELISA-positive rate was 14.69%. The ELISA-positive rates varied significantly, with the lowest seroprevalence in July (7.14%, 95% CI = 3.32–13.13) and highest in September (19.74%, 95% CI = 14.83–25.44). NT antibodies were detected in 2.2% of dogs, with 69.56% of samples collected in December, suggesting that most of the animals were exposed at the end of the study period since it was in the midst of the second wave. 

More serologically positive dogs made it possible to determine the risk factors for SARS-CoV-2 infection in dogs. Sex, breed and age were identified as significant risk factors for SARS-CoV-2 seroconversion. Male dogs were at increased risk of contracting the infection. In contrast, dogs under one year of age seemed to be less susceptible to SARS-CoV-2 infection [19]. In men, initial studies of human infections showed higher COVID-19 susceptibility, severity and fatality [28,29]. On the other hand, children and adolescents seem to be at a lower risk of contracting the infection [30]. Biological, psychological, behavioral and social factors may put specific gender or age groups at disproportionate risk of infection in the human population [31]. Since there is no evidence of SARS-CoV-2 infection independently spreading in the dog population, sex- and age-dependent difference in susceptibility to SARS-CoV-2 infection in both species seem to result from the intrinsic biological factors described for other diseases [32,33,34,35,36]. 

In addition, cat serum samples were collected in the Veterinary Teaching Hospital during December 2020, the same period when dog and human serum samples were collected in the two previously mentioned studies [16,27]. As determined by ELISA, there was no significant difference in seroprevalence in the human, dog and cat population in Zagreb during the second wave (Table 3). It is clear that, as in dogs, SARS-CoV-2 infection was widespread in the cat population and comparable to the seroprevalence in humans. 

## 4. COVID-19 in Wildlife

One published study analyzed the prevalence of COVID-19 in wildlife. From June 2020 to February 2021, blood, muscle extract and fecal samples of free-living wild boars (*Sus scrofa*), red foxes (*Vulpes vulpes*) and jackals (*Canis aureus*), and blood and cloacal swabs of yellow-legged gulls (*Larus michahellis*) were tested for SARS-CoV-2 (Table 4). The study also included fecal samples from zoo animals. Although some blood samples and muscle extracts gave positive ELISA results, all tested negative by surrogate VNT. The same was true for the RT-PCR results, and none of the fecal samples tested positive, giving no evidence of spillover of SARS-CoV-2 to free-living or captive wild animals [37].

## 5. SARS-CoV-2 in the Environment

### 5.1. SARS-CoV-2 in Households with COVID-19 Cases

The transmission of SARS-CoV-2 from contaminated surfaces or fomites has been a concern during the COVID-19 pandemic. Households have been important sites of SARS-CoV-2 transmission due to prolonged contact with an infected person and environmental contamination in these settings [38].

To analyze the extent of the environmental contamination with SARS-CoV-2, during the first wave of the COVID-19 pandemic in Croatia (April–September 2020), environmental samples were tested for the presence of SARS-CoV-2 using qRT-PCR. One hundred and seventy-one environmental samples were taken from the environment of patients with confirmed COVID-19 in 15 non-healthcare settings (Table 5). The sampling sites were: telephone/cellular phone, keyboard, light switch, thermometer, TV remote, door handle, pillowcase, toilet seat, fridge handle, etc. Sampling was performed before surface cleaning. Between 8 and 15 samples were collected from each setting, with a mean of 11.4 per location. Surface samples were taken in the interval between the 1st and 19th day of a patient’s positive test (positive SARS-CoV-2 RT-PCR test, but no data for patient cycle threshold; Ct value). SARS-CoV-2 RNA detection rates varied from 0% to 37.5%. Most of the Ct values were ≥30 (30–39), which assumes a low viral load. The investigation confirmed that 9/15 (60.0%) of the tested locations were contaminated with SARS-CoV-2, with one or more positive samples per location. Twenty-three individual sites had positive detection of SARS-CoV-2 RNA (13.5%) (Table 5). High-touch personal objects were most frequently contaminated (17.3%; 95% CI = 8.2–30.3), followed by surfaces in patients’ rooms (14.6%; 95% CI = 5.6–29.2), kitchens (13.3%, 95% CI = 3.8–30.7) and bathrooms (8.3%, 95% CI = 2.3–19.9).

Although SARS-CoV-2 was detected on inanimate surfaces in households with COVID-19 cases, the SARS-CoV-2 RNA quantification data (high Ct values) suggest that the risk of SARS-CoV-2 infection via fomite transmission was low. Similar results were observed in other studies [39]. 

### 5.2. SARS-CoV-2 in Wastewater

Wastewater-based epidemiology (WBE) is a tool to monitor the presence/circulation of biological or chemical agents in a population [40,41]. The detection of SARS-CoV-2 in the urine and feces of patients with symptomatic and asymptomatic infection [42,43] implies that the virus may be detected in the wastewater [44,45,46]. Several studies have shown that SARS-CoV-2 RNA dynamics in raw wastewater coincide with the dynamics of COVID-19 cases [45,47]. WBE can inform of the presence of the virus, viral dynamics and the emergence of new viral variants [48]. Therefore, wastewater testing can be used as an early warning tool for virus circulation in the population [44]. 

**Figure 2:**
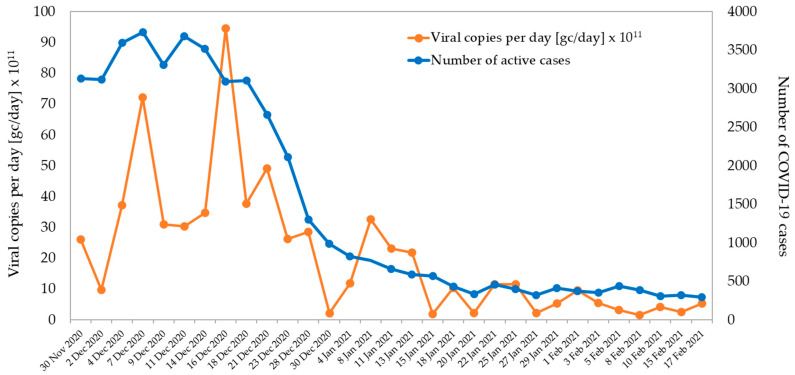
SARS-CoV-2 viral load in wastewater at the entrance to the Central Wastewater Treatment Plant of the City of Zagreb (December 2020–February 2021).

As a part of the WHO project, from December 2020 to February 2021, a wastewater SARS-CoV-2 monitoring was performed in Zagreb, two times per week at the location Zagreb Wastewater Treatment Plant (WWTP). Sixty-two 24 h composite samples were analyzed (31 of affluent and 31 of effluent) for SARS-CoV-2 RNA. The RT-PCR results showed the presence of SARS-CoV-2 RNA in 30 affluent water samples (96.8%) that reached the WWTP (Figure 2). The SARS-CoV-2 RNA load ranged from 9.7 × 10^11^–9.5 × 10^12^ copies/day (1–21 December 2020) to 1.9 × 10^11^–2.2 × 10^12^ copies/day (13 January–17 February 2021). All effluent water samples tested negative. During the same period, the active number of reported COVID-19 cases in the city of Zagreb was decreasing, with the highest number of active cases (N = 3731) observed on 6 December 2020 and the lowest (N = 291) observed on 17 February 2021.

The data obtained during SARS-CoV-2 monitoring in wastewater showed low virus prevalence in wastewater. These results were in accordance with the epidemiological situation in the first two weeks of February 2021. 

## 6. SARS-CoV-2 Genetic Diversity in Croatia

SARS-CoV-2 is continuously evolving and adapting to the environment. Therefore, new genetic variants have appeared throughout the pandemic. They are classified as variants of interest (VoIs), concern (VoCs) and under monitoring. The most important VoCs include B.1.351 (Beta), P.1 (Gamma) and the currently dominant B.1.617.2 (Delta), while the circulation of B.1.1.7 variant (Alpha) has been drastically reduced. Genomic surveillance of SARS-CoV-2 is essential to detect, monitor and assess virus variants that can increase transmissibility and disease severity [49,50]. 

**Figure 3:**
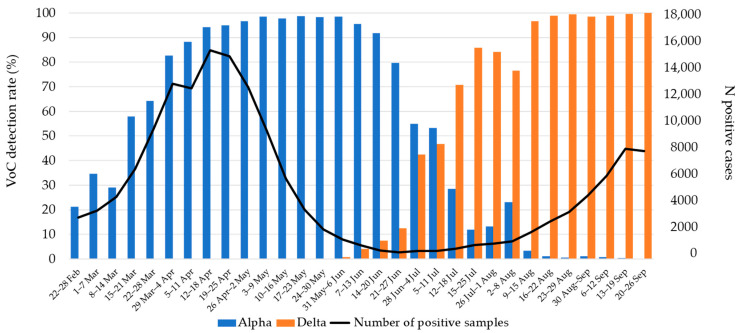
Prevalence of SARS-CoV-2 Alpha and Delta VoCs detected during the third and fourth epidemic wave in Croatia (February–September 2021).

To monitor the prevalence and spread of VoCs, whole-genome sequencing (WGS) of SARS-CoV-2 positive samples in Croatia was conducted weekly from February 2021. By the 26 October 2021, 11,280 samples were processed and 8874 samples were successfully sequenced. During the first and second epidemic waves, SARS-CoV-2 lineages from clades G, GR and GV were dominant. A marked weekly increase in the Alpha variant from the GRY clade indicated the beginning of the third epidemic wave in Croatia. The Alpha variant was dominant from the beginning of March 2021 (>50% of positive samples). In the first week of June 2021, the first Delta variants were detected, rapidly replacing Alpha variants and reaching by the beginning of August 2021 more than 90% of all sequenced samples (Figure 3). Beta and Gamma variants detected in a low number of samples were closely related to traveling abroad and were not transmitted locally [51].

## 7. Conclusions

The COVID-19 pandemic has reinforced the importance of an integrated ‘One Health’ approach for the surveillance of zoonotic diseases [52]. The seroprevalence rates were significantly higher in all studied population groups after the second pandemic wave in Croatia. Furthermore, population groups at higher risk of infection, risk factors and epidemiology have changed in the second wave. It is still to be determined to what extent these changes result from human activities or virus evolution, highlighting the need for continuous SARS-CoV-2 surveillance in the future. This will enable the implementation of the efficient measures to protect population groups at increased risk of infection, especially those particularly vulnerable to COVID-19. Environmental studies have proven to be very useful as a surveillance tool. SARS-CoV-2 was detected on inanimate surfaces in the households of COVID-19 patients; however, the viral load was low, indicating that the environment does not seem to pose a high risk for SARS-CoV-2 transmission. The SARS-CoV-2 RNA dynamics in the wastewater coincided with the dynamics of human COVID-19 cases and can be used to estimate the number of infected people in some areas. Finally, in addition to human cases, SARS-CoV-2 infections were confirmed in pet animals in Croatia. High seroprevalence in companion animals that are in close contact with their owners is another major difference in SARS-CoV-2 epidemiology compared to closely related and, in many other senses, similar viruses, such as SARS-CoV and MERS-CoV. The detection of SARS-CoV-2 in humans, animals and the environment suggests that the ‘One Health’ approach is critical to controlling COVID-19 and future pandemics.

## Figures and Tables

**Table 1:** Seroprevalence of SARS-CoV-2 in different population groups in Croatia.

Population Group	Sampling Time	N Tested	SARS-CoV-2 IgG ELISA		SARS-CoV-2 VNT		Reference
			N (%)	95% CI	N (%)	95% CI	
First pandemic wave							
Industry workers	April 2020	1494	19 (1.27) *	0.77–1.98	NT	NT	[12]
Healthcare workers	April–May 2020	592	16 (2.7)	1.5–4.3	9 (1.5)	0.7–2.9	[13]
Children and adolescents	May 2020	240	9 (3.9)	1.7–7.0	7 (2.9)	1.2–5.9	[14]
Hemodialysis patients	May 2020	136	9 (6.6)	3.1–12.1	0 (0)	0–2.7 **	[15]
Veterinary personnel	May 2020	122	6 (4.9)	1.8–10.4	0 (0)	0–2.9 **	[15,16]
Construction workers	May–June 2020	135	4 (2.9)	0.8–7.4	3 (2.2)	0.4–6.4	[15]
General population	May–July 2020	1088	24 (2.2)	1.4–3.2	2 (0.2)	0.02–0.7	[17]
Professional athletes	June 2020	90	10 (11.1)	5.5–19.5	5 (5.5)	1.8–12.5	[15]
Second pandemic wave							
Liver transplant recipients	September–November 2020	280	59 (21.1)	16.4–26.3	10 (3.6)	1.7–6.5	[18]
Kidney transplant recipients	September–November 2020	232	44 (19.0)	14.1–24.6	6 (2.6)	0.9–5.5	[18]
Children and adolescents	October–November 2020	308	27 (8.8)	5.0–12.5	26 (8.4)	5.6–12.1	[14]
General population	December 2020–February 2021	1436	360 (25.1)	22.8–27.4	268 (18.7)	16.7–20.8	[17]
Veterinary personnel	March 2021	121	22 (18.2)	11.8–26.2	11 (9.1)	4.6–15.7	[15,16]

IgG = immunoglobulin G; ELISA = enzyme-linked immunosorbent assay; VNT = virus neutralization test; CI = confidence intervals; * Immunochromatography test (ICT); NT = not tested; ** one-sided 97.5% CI.

**Table 2:** Risk analysis for SARS-CoV-2 seropositivity.

Population Group	SARS-CoV-2 IgG ELISA			SARS-CoV-2 VNT		
	OR	95% CI OR	*p*	OR	95% CI OR	*p*
First pandemic wave						
General population	Ref.			Ref.		
Industry workers *	0.57	0.31–1.05	0.07	NA	NA	NA
Healthcare workers	1.23	0.65–2.34	0.52	8.38	1.8–38.20	0.002
Children and adolescents	1.73	0.79–3.76	0.16	16.31	3.37–79.30	<0.001
Hemodialysis patients	3.14	1.43–6.91	0.007	1.59	0.08–33.33	1.00
Veterinary personnel	2.29	0.92–5.72	0.11	1.77	0.08–37.16	1.00
Construction workers	1.35	0.46–3.96	0.54	12.34	2.04–74.53	0.01
Professional athletes	5.54	2.56–11.99	<0.001	31.94	6.11–167.09	<0.001
Second pandemic wave						
General population	Ref.			Ref.		
Liver transplant recipients	0.80	0.58–1.09	0.15	0.16	0.08–0.31	<0.001
Kidney transplant recipients	0.70	0.49–0.99	0.04	0.12	0.05–0.26	<0.001
Children and adolescents	0.29	0.19–0.43	<0.001	0.40	0.16–0.61	<0.001
Veterinary personnel	0.66	0.41–1.07	0.09	0.44	0.23–0.82	0.008

IgG = immunoglobulin G; ELISA = enzyme-linked immunosorbent assay; VNT = virus neutralization test; OR = odds ratio; * Immunochromatography test (ICT); CI = confidence intervals.

**Table 3:** Seroprevalence of SARS-CoV-2 in humans and pet animals in Zagreb, December 2020.

Sample Origin	SARS-CoV-2 IgG ELISA			OR	95% CI OR	*p*
	N Tested	N Positive (%)	95% CI			
Human	458	94 (20.5)	16.92–24.52	Ref.	–	–
Dog	167	31 (18.6)	12.97–25.30	0.88	0.56–1.39	0.59
Cat	29	4 (13.8)	3.89–31.66	0.62	0.21–1.82	0.38

IgG = immunoglobulin G; ELISA = enzyme-linked immunosorbent assay; CI = confidence intervals; OR = odds ratio.

**Table 4:** Seroprevalence of SARS-CoV-2 in free-living wild animals.

Animal Species	Sampling Time	N Tested	SARS-CoV-2 IgG ELISA		SARS-CoV-2 sVNT		SARS-CoV-2 RT-PCR
			N (%)	95% CI	N (%)	95% CI	N (%)
Yellow-legged gulls (*Larus michahellis*)	November 2020	111	0 (0)	0–3.3 *	0 (0)	0–3.3 *	0 (0)
Wild boars (*Sus scrofa*)	June–December 2020	153	6 (3.9)	1.5–8.3%	0 (0)	0–2.4 *	0 (0)
Red foxes (*Vulpes vulpes*)	June–November 2020	204	6 (2.9)	1.0–6.2	0 (0)	0–1.8 *	0 (0)
Jackals (*Canis aureus moreoticus*)	June–October 2020	65	3 (4.6)	0.9–12.9	0 (0)	0–5.5 *	0 (0)

IgG = Immunoglobulin G; ELISA = enzyme-linked immunosorbent assay; sVNT = surrogate virus neutralization test; RT-PCR = reverse transcriptase-polymerase chain reaction; * one-sided 97.5% confidence interval.

**Table 5:** Detection of SARS-CoV-2 in the household settings of COVID-19 human cases.

Sampling Location	High-Touch Personal Objects ^1^	Room ^2^	Toilet/Bathroom ^3^	Kitchen ^4^	Total		
	N Positive/N	N Positive/N	N Positive/N	N Positive/N	N Positive/N	% Positive	95% CI
1	0/3	0/5	0/3	-	0/11	0	0–28.4 *
2	0/2	1/1	1/4	1/4	3/11	27.2	6.0–60.1
3	0/3	0/3	0/5	0/3	0/14	0	0–25.2 *
4	1/3	1/4	0/2	0/2	2/11	18.2	2.3–51.8
5	2/4	2/4	0/3	0/1	4/12	33.3	9.9–65.1
6	0/6	0/3	0/5	-	0/14	0	0–23.2 *
7	0/4	0/3	1/3	0/1	1/11	9.1	2.3–49.3
8	0/2	2/2	0/1	1/3	3/8	37.5	8.5–75.5
9	0/2	0/4	0/2	0/1	0/9	0	0–33.6 *
10	3/5	0/1	0/2	1/3	4/11	36.4	10.9–69.2
11	0/5	0/3	0/1	-	0/9	0	0–33.6 *
12	0/4	0/2	0/3	0/3	0/12	0	0–26.5 *
13	0/3	0/4	0/5	1/2	1/14	0	0–33.9
14	1/2	0/1	0/3	0/3	1/9	1.1	2.8–48.2
15	2/4	0/1	2/6	0/4	4/15	26.7	7.8–55.1
Total	9/52 (17.3%)	6/41 (14.6%)	4/48 (8.3%)	4/30 (13.3%)	23/171	13.5	8.7–19.5

^1^ Telephone/cellular phone, keyboard, light switch, thermometer, TV remote, reading glasses, etc.; ^2^ Door handle, window handle, chair, pillow, etc.; ^3^ Light switch, toilet seat, faucet, toilet brush etc.; ^4^ Locker, fridge handle, light switch, fridge handle, etc.; CI = confidence intervals; * one-sided 97.5% CI.

## Data Availability

Not applicable.
